# Investigation of the Etching Resistance of Yttrium Oxyfluoride Coating Deposited via Atmospheric Plasma Spraying Against Cl_2_/O_2_ Plasma

**DOI:** 10.3390/ma18091903

**Published:** 2025-04-23

**Authors:** Zaifeng Tang, Yukun Lv, Kaiqu Ang, Bing Wang, Xiaojun Jiang, Yuwei Wang, Jin Xu, Hua Meng, Hongli Chen, Ying Shi, Linjun Wang

**Affiliations:** 1 School of Materials Science and Engineering, Shanghai University, 333 Nanchen Road, Shanghai 200444, China; zaifeng_81@163.com (Z.T.); yshi@shu.edu.cn (Y.S.); 2 Shanghai Huali Integrated Circuit Corporation, 6 Liangteng Road, Pudong New Area, Shanghai 201314, China; lvyukun0106@163.com (Y.L.); akq722@163.com (K.A.); colinwang0105@163.com (Y.W.); mrxujin@yeah.net (J.X.); mhuacai@163.com (H.M.); 3 Chongqing Genori Technology Co., Ltd., No. 66, Sendi Avenue, Xipeng Town, Jiulongpo District, Chongqing 401326, China; wangbing@genori.com.cn (B.W.); jiangxiaojun@genori.com.cn (X.J.); chenhongli@genori.com.cn (H.C.); 4 Zhejiang Institute of Advanced Materials, Shanghai University, Jiashan 314113, China

**Keywords:** Cl_2_/O_2_ plasma, etching, yttrium oxyfluoride (YOF), atmospheric plasma spraying (APS)

## Abstract

Chlorine-based plasma is widely used in key etching applications. However, while etching the wafer materials, chlorine plasma can cause damage to the internal components of the etching chamber, which adversely affects the equipment’s lifespan. As a result, selecting appropriate coating materials for the chamber’s internal components is essential for mitigating corrosion. The etch resistance of these coatings directly impacts not only the quality of wafer production but also the operational safety and maintenance cycle of the etching equipment. In this study, three yttrium oxyfluoride coatings with different oxygen contents (3%, 6%, and 9%) were prepared using atmospheric plasma spraying technology. The etch resistance of these YOF coatings, as well as yttrium oxide coating, was systematically investigated under a Cl_2_/O_2_ plasma environment. Transmission electron microscopy analysis revealed that at the initial stage, Cl^−^ formed a protective layer on the surface of the YOF coatings, effectively slowing down further etching by Cl^−^. Among the samples, the YOF 6% coating exhibited the best etching resistance, which is primarily attributed to its higher capacity for Cl^−^ adsorption. Overall, YOF coatings demonstrated excellent resistance in chlorine-based plasma environments, with YOF 6% in particular showing great potential as an ideal protective material for etching chamber components.

## 1. Introduction

Modern computer chips are manufactured through a series of complex processes carried out on silicon wafers, involving the use of a wide range of advanced semiconductor equipment. For state-of-the-art chips, the number of processing steps can reach hundreds or even thousands of steps, covering material processing, metrology, visual inspection, and electrical testing [1,2,3,4,5]. To enhance chip performance and yield, these processes are meticulously co-optimized and, to a large extent, built upon the experience from previous technology nodes, thereby accelerating the time-to-market for advanced node products.

Plasma etching is a crucial process in semiconductor manufacturing [6,7,8]. In the semiconductor industry, plasmas containing active compounds such as fluorine and chlorine (e.g., CF_4_, C_4_F_6_, Cl_2_, HBr, SF_6_) are commonly used to etch silicon (Si), silicon dioxide (SiO_2_), and other materials [9]. The ion bombardment and reaction radiation from the plasma not only form the desired patterns on the wafer surface but also etch the chamber wall surfaces exposed to the plasma [10,11]. During the etching process, these gases react with the ceramic coating inside the chamber, leading to the corrosion of the etching machine components. The particles generated will contaminate the silicon wafer, fall onto its surface, and cause defects, significantly reducing the yield [12,13]. Therefore, the study of ceramic materials with high hardness, corrosion resistance, and chemical stability for covering the inner walls of the etching chamber is key to improving the yield of etching products [14,15,16,17,18].

Among the various plasma etching-resistant coating materials, ceramic materials are widely recognized for their low etching rate and low chemical reactivity, with yttrium oxide (Y_2_O_3_) being particularly notable [19,20,21,22,23]. Although yttrium oxide offers certain advantages as an etching-resistant material, it also has some drawbacks, such as its relatively weak resistance to fluorinated gases and lower chemical stability compared with other ceramic materials like YOF (e.g., the formation enthalpy of the metal–oxygen bond in YOF is 392 kJ·mol^−1^, lower than Y_2_O_3_’s 318 kJ·mol^−1^) [24,25]. In recent years, yttrium fluoride (YOF) has gained significant attention as an emerging etching-resistant ceramic coating material, due to its ability to effectively suppress chemical reactions with fluorinated gases such as CF_4_ and NF_3_ [26,27,28,29,30,31,32]. The atmospheric plasma spraying (APS) technique is commonly used to coat parts with ceramic materials like Y_2_O_3_ and YOF, due to its high spraying efficiency, dense coating structure, strong bond strength between the coating and substrate, wide availability of spraying materials, and low processing costs. By using the APS method, it is possible to quickly form the desired coating on the substrate by melting ceramic powders with particle sizes ranging from tens of microns to smaller sizes in a high-temperature plasma (10,000 K or higher).

The chlorine plasma, due to its mild reactions and moderate etching rate, enhances the controllability of the etching process, making it widely used in critical etching applications. However, previous studies on the etch resistance of YOF coatings have primarily focused on fluorine-based plasma environments [30,33], with limited research on the performance of YOF coatings in chlorine-based plasma environments [34]. This paper investigates the etching behavior of Y_2_O_3_ and YOF coatings deposited on Al_2_O_3_ substrates via atmospheric plasma spraying (APS) in Cl_2_/O_2_ plasma. The etch resistance of the coatings is evaluated by analyzing changes in their microstructure and chemical composition. The results show that the YOF coating produced from raw material with 6% oxygen content exhibits excellent etch resistance in Cl_2_ plasma. Further research reveals that the oxygen content in the YOF raw material plays a crucial role in modulating the etching behavior and defect chemistry of the YOF coatings. Additionally, this paper evaluates the potential of YOF coatings as protective coatings for etching chamber walls.

## 2. Experimental Materials and Methods

### 2.1. Experimental Materials

YOF powders purchased from Nippon Yttrium Co., Ltd. (Fukuoka, Japan) and Y_2_O_3_ ceramic powders purchased from Shin-etsu (Tokyo, Japan) were used as raw materials for preparing high-performance etch-resistant coatings. The coatings prepared from raw materials with different oxygen contents (oxygen mass fractions of 3%, 6%, and 9%) were labeled as YOF 3%, YOF 6%, and YOF 9%, respectively. Additionally, Y_2_O_3_ was used as a reference material. The deionized water used in the experiments was prepared in the laboratory, and ethanol, acetone, and other chemical reagents were purchased from Sinopharm without further purification.

### 2.2. Experimental Methods

The YOF series coatings and Y_2_O_3_ coatings were prepared using the Oerlikon Metco APS system from the Oerlikon Metco, New York, NY, USA. This APS system generates a high-temperature plasma jet by ionizing a mixture of argon and hydrogen gases, which melts the ceramic particles. The molten particles are then sprayed onto anodized aluminum substrates (50 mm × 50 mm × 3 mm). By precisely controlling the process parameters, coatings with a thickness of 200 µm were successfully formed on the metal substrates. To ensure the cleanliness of the aluminum alloy substrates, they were first sandblasted with SiO_2_ and then cleaned with acetone. To test the etching resistance of the coatings, the Kiyo EX inductively coupled plasma (ICP) etching machine from Lam Research was used in this study. The specific steps of the experiment are as follows: Cl_2_, O_2_, and He gases were introduced into the etching machine’s reaction chamber at flow rates of 200 standard cubic centimeters per minute (sccm), 10 sccm, and 15 sccm, respectively, while maintaining a constant pressure of 10 mTorr. The TCP RF power was set to 350 W, with an RF bias voltage of 250 V, and the plasma exposure time for the coating substrate was 300 h. To characterize the raw materials Y_2_O_3_ and YOF powders used for the coatings, field emission scanning electron microscopy (FE-SEM, SU8010, Hitachi, Tokyo, Japan) was employed. Cross-sectional, surface morphology, and microstructural analyses of the coating samples and the Cl_2_ plasma-etched samples were conducted using scanning electron microscopy (SEM5000, CIQTEK Co., Ltd., Hefei, China). Additionally, the phase composition of the raw materials and coatings was analyzed using X-ray diffraction (XRD, Rigaku Ultima IV, Tokyo, Japan; radiation source: Cu-Kα (λ = 1.5418 Å); scan range: 10–80^∘^ (2θ); scan rate: 2^∘^/min). The elemental composition of the coating surface was analyzed using X-ray photoelectron spectroscopy (XPS, K-Alpha, Thermo Scientific, Waltham, MA, USA, monochromatic Al-Kα X-ray source, voltage 100 V, spot size 450 µm, energy 50 eV, detection angle 45^∘^). The crystal structure of the coatings was further examined using transmission electron microscopy (TEM). At the same time, the surface roughness of the coatings was measured using an atomic force microscope (AFM, Bruker Icon SCANASYST-6AIR, Billerica, MA, USA) to comprehensively evaluate the microstructure and surface characteristics of the coatings.

## 3. Discussion and Results

**Figure 1:**
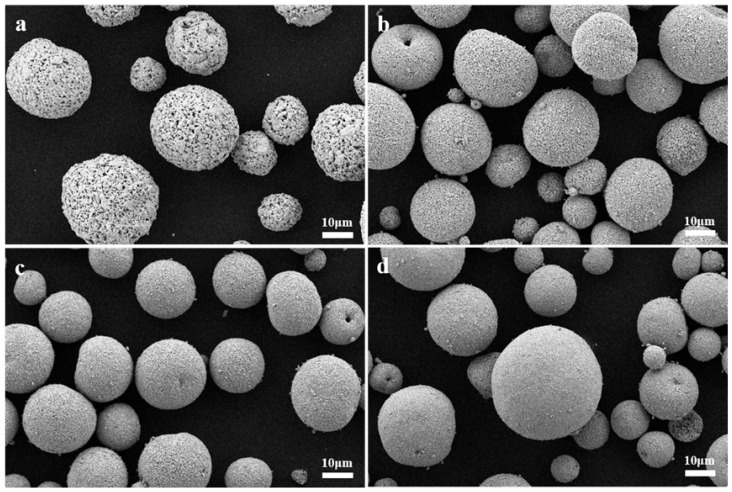
SEM images of (**a**) Y_2_O_3_, (**b**) YOF 3%, (**c**) YOF 6%, and (**d**) YOF 9% powders.

The Y_2_O_3_ and YOF powder raw materials were analyzed using scanning electron microscopy, and the results are shown in Figure 1. All of the raw materials consist of spherical particles. Particle size has an important impact on the spraying performance of the final coating, making it a key physical characteristic in the powder coating manufacturing process. Therefore, particle size and repose angle were further characterized using a particle size analyzer, with the results being shown in Table 1. The Y_2_O_3_ and YOF powders exhibit typical particle size distributions, with average particle sizes of 34.0 µm, 27.1 µm, 26.6 µm, and 27.7 µm and repose angles of 33.5^∘^, 33.7^∘^, 31.4^∘^, and 30.6^∘^, respectively. The spherical structure and limited particle size distribution of the raw material powders indicate that they are suitable for plasma spraying, providing good materials for the subsequent preparation of ceramic coatings through atmospheric plasma spraying.

**Figure 2:**
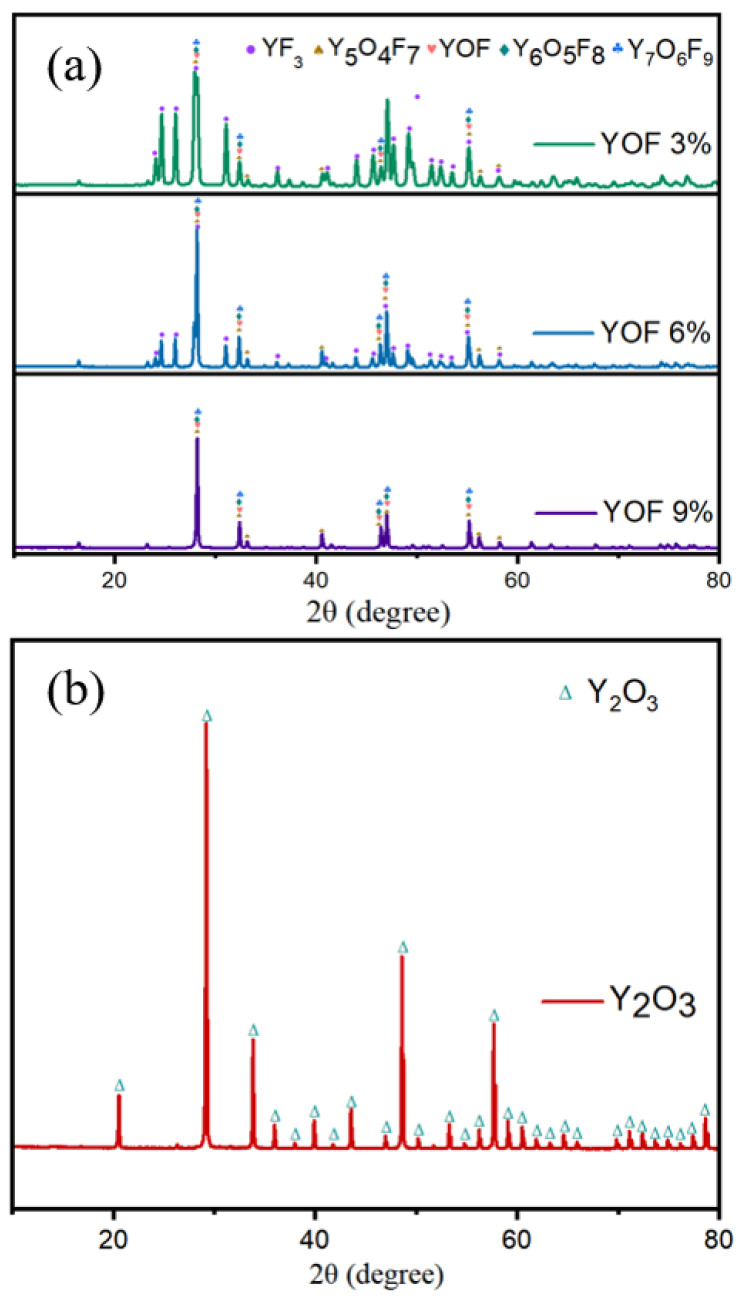
XRD patterns of (**a**) YOF powders and (**b**) Y_2_O_3_ powder.

The phase analysis of the raw materials was conducted using XRD, with the results being shown in Figure 2a. The analysis reveals that the XRD patterns of the YOF raw materials are consistent with the standard data for YOF (JCPDS No. 71-2100), Y_2_O_3_ (JCPDS No. 411105), and YF_3_ (JCPDS No. 74-0911). Additionally, the YOF powders are not composed of a single YOF phase, but rather a mixture of multiple YOF phases, such as Y_5_O_4_F_7_, Y_6_O_5_F_8_, and Y_7_O_6_F_9_. The YF_3_ peak is detectable in all YOF powders due to incomplete reactions, but its low concentration allows it to be considered negligible in terms of experimental impact. Figure 2b shows that the Y_2_O_3_ powder contains only the Y_2_O_3_ phase.

**Figure 3:**
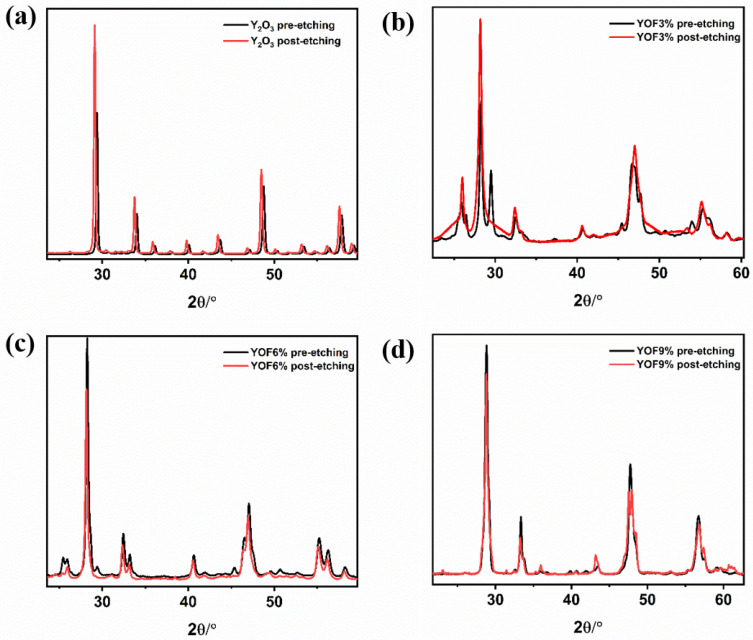
XRD spectra of the (**a**) Y_2_O_3_ coatings as-deposited and after plasma etching, (**b**) YOF 3% coatings as-deposited and after plasma etching, (**c**) YOF 6% coatings as-deposited and after plasma etching, and (**d**) YOF 9% coatings as-deposited and after plasma etching.

XRD was further used to characterize the phase composition of the coatings before and after etching, and the results are shown in Figure 3. The analysis indicates that after etching with chlorine plasma, the XRD diffraction peaks of all coatings shifted to the left. Taking the highest diffraction peak of each coating as an example, the diffraction peak of Y_2_O_3_ shifted from 29.38^∘^ to 29.12^∘^, YOF 3% shifted from 28.27^∘^ to 28.18^∘^, YOF 6% shifted from 28.24^∘^ to 28.14^∘^, and YOF 9% shifted from 28.84^∘^ to 28.78^∘^. According to the Bragg diffraction formula, the leftward shift of the diffraction peak indicates an increase in the lattice constant, which typically suggests a change in the atomic spacing within the lattice. In this study, the leftward shift of the diffraction peaks is primarily attributed to the high-energy Cl^−^ from the chlorine plasma bombarding the surface of the coatings during etching. Specifically, the radius of Cl^−^ is 0.181 nm, which is larger than that of F^−^ (0.133 nm) and O^2−^ (0.14 nm). Therefore, during etching, Cl^−^ is likely to replace some of the F^−^ and O^2−^ on the coating surface, leading to changes in the coating’s lattice structure and causing a shift of the diffraction peaks. This phenomenon further supports the idea that Cl^−^ interacts with the coating material during etching and affects its lattice structure.

**Figure 4:**
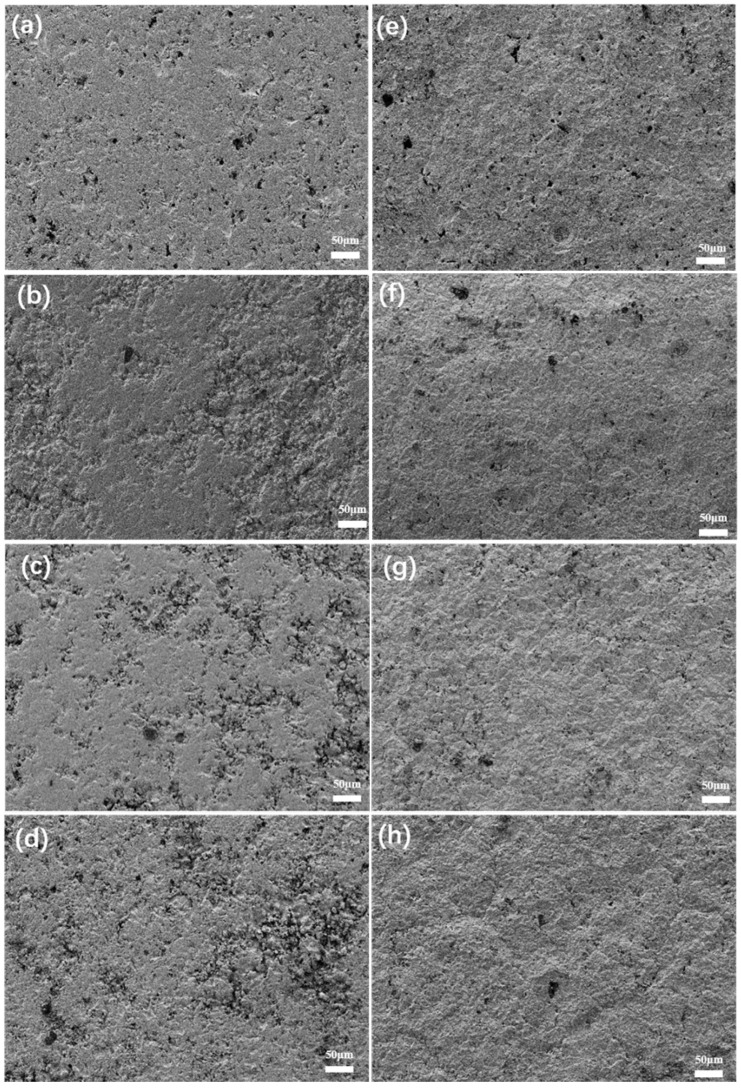
SEM images of (**a**) Y_2_O_3_, (**b**) YOF 3%, (**c**) YOF 6%, and (**d**) YOF 9% coatings before etching, and SEM images of (**e**) Y_2_O_3_, (**f**) YOF 3%, (**g**) YOF 6%, and (**h**) YOF 9% coatings after etching.

Figure 4a–d show scanning electron microscope (SEM) images of the surface of Y_2_O_3_ and YOF coatings prepared by the atmospheric plasma spraying (APS) method. It can be observed that the surface morphology of all coatings consists of smooth and rough areas. The smooth areas are formed by molten particles that solidify on the substrate surface after being splattered and dispersed, while the rough areas are composed of particles that were not fully melted. These particles did not fully melt and bond with other particles, resulting in a rougher surface. To further observe the changes in the coating after chlorine plasma etching, the coatings were subjected to etching treatment, and the surface morphology after etching is shown in Figure 4e–h. By comparing the microscopic morphology before and after etching, it can be seen that both YOF series coatings and Y_2_O_3_ coatings underwent significant changes after chlorine plasma etching. Specifically, the YOF series coatings exhibited a more compact surface structure after etching, while the surface structure of the Y_2_O_3_ coating appeared more loose. This indicates that YOF coatings have better etching resistance compared with Y_2_O_3_ coatings.

**Figure 5:**
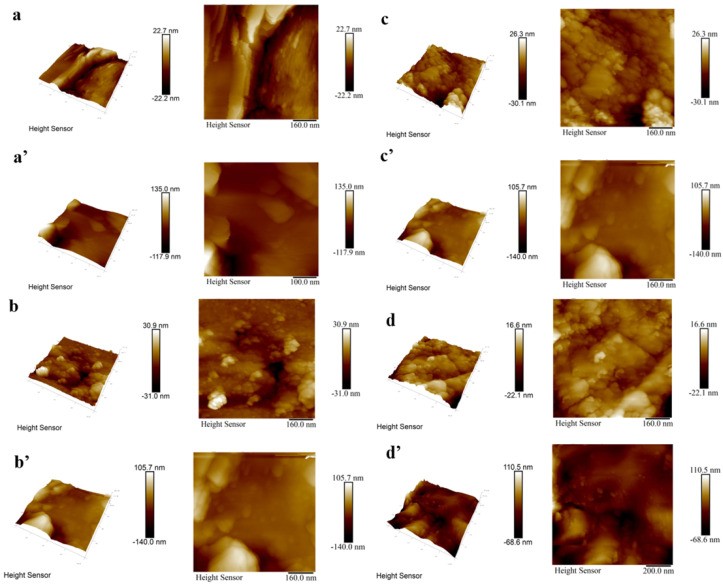
Atomic force microscopy (AFM) images of (**a**) Y_2_O_3_ coating before etching, (**b**) YOF 3% coating before etching, (**c**) YOF 6% coating before etching, and (**d**) YOF 9% coating before etching; and AFM images of (**a’**) Y_2_O_3_ coating after etching, (**b’**) YOF 3% coating after etching, (**c’**) YOF 6% coating after etching, and (**d’**) YOF 9% coating after etching.

Additionally, Figure 5 presents the changes in roughness of all coatings before and after etching, measured by atomic force microscopy (AFM). Before etching, the surface roughness of Y_2_O_3_, YOF 3%, YOF 6%, and YOF 9% coatings was 5.0 nm, 4.7 nm, 4.9 nm, and 6.2 nm, respectively; after etching, the roughness values increased to 19 nm, 18.5 nm, 18.6 nm, and 15.9 nm, respectively. This indicates that the surface roughness of the coatings increased after etching, which may be attributed to the bombardment of the coating surface by Cl_2_ plasma, leading to material loss and structural damage. It is worth noting that the surface roughness of Y_2_O_3_ coatings is higher than that of YOF coatings. This is likely due to the larger particle size of Y_2_O_3_ powder. During the APS coating process, larger particles are more difficult to fully melt, and unmelted particles impact the substrate and break apart, leading to more defects on the coating surface and thus increasing the surface roughness. Especially under the etching effect of chlorine plasma, the regions with unmelted particles are more prone to detachment, further exacerbating the increase in surface roughness of the coating.

**Figure 6:**
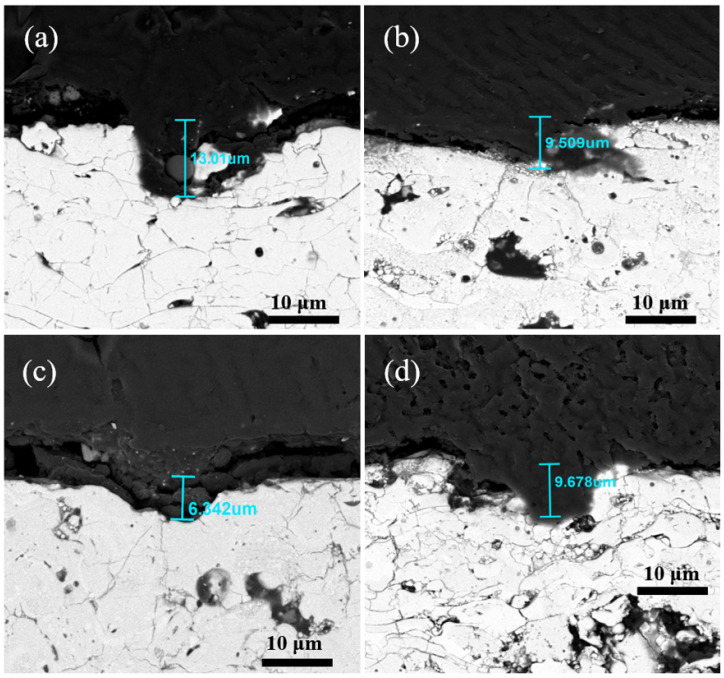
SEM images for the etching depth of (**a**) Y_2_O_3_, (**b**) YOF 3%, (**c**) YOF 6%, and (**d**) YOF 9% coatings.

This study carefully investigated the etching resistance of Y_2_O_3_, YOF 3%, YOF 6%, and YOF 9% coatings in chlorine plasma. During the testing process, a masking method was employed. A portion of the coating surface was masked to prevent this area from being affected by the chlorine plasma during etching. The masked region of the coating surface remained unetched, while the unmasked region was exposed to the etching environment. By comparing the surface height difference between the masked and unmasked regions after etching, the etching resistance of the coatings was effectively evaluated. The etching depth was characterized using scanning electron microscopy (SEM). Figure 6 shows the surface height differences after etching for Y_2_O_3_, YOF 3%, YOF 6%, and YOF 9% coatings, which were 13.01 µm, 9.509 µm, 6.342 µm, and 9.678 µm, respectively. The results indicate that the etching depth of the YOF series coatings is generally smaller than that of the Y_2_O_3_ coating, suggesting that the YOF series coatings have better etching resistance than the Y_2_O_3_ coating. Among the YOF series coatings, the YOF 6% coating exhibited the shallowest etching depth, demonstrating the best etching resistance. Additionally, the etching performance of the coatings was further evaluated through mass change measurements. Table 2 shows the weight loss of the four coatings after etching. All coatings experienced some degree of weight reduction after etching, with the specific weight loss per unit area being as follows: 0.486 g/cm^2^ for Y_2_O_3_, 0.127 g/cm^2^ for YOF 3%, 0.018 g/cm^2^ for YOF 6%, and 0.117 g/cm^2^ for YOF 9%. It can be observed that the YOF 6% coating had the lowest weight loss per unit area, further confirming its superior resistance to chlorine plasma etching. Based on the above test results, the YOF 6% coating exhibited the best etching resistance, with the shallowest etching depth and the smallest weight loss compared with the other coatings. Therefore, the YOF 6% coating may offer stronger etching resistance in practical applications, especially in environments with high-intensity plasma exposure.

**Figure 7:**
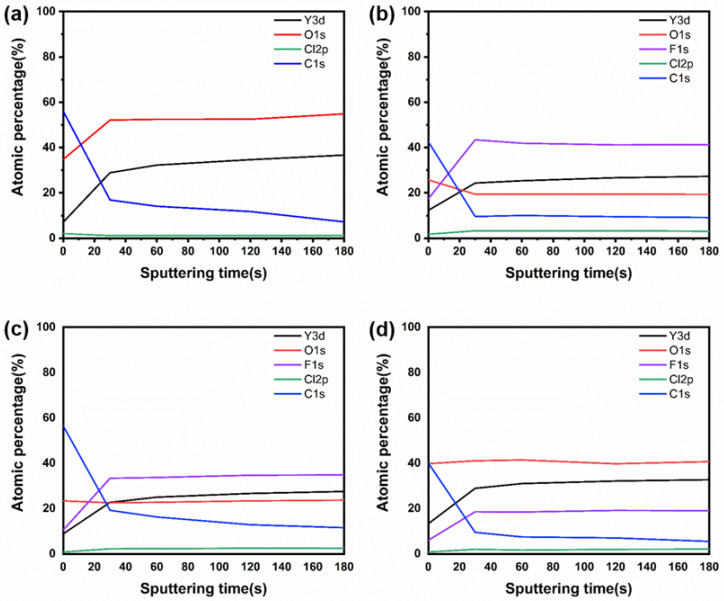
Variations in chemical compositions measured with XPS with the sputtering time for (**a**) Y_2_O_3_ coating, (**b**) YOF 3% coating, (**c**) YOF 6% coating, and (**d**) YOF 9% coating after exposure to Cl_2_/O_2_ plasma.

An XPS analysis was conducted to investigate the chemical composition changes of Y_2_O_3_, YOF 3%, YOF 6%, and YOF 9% coatings during the Cl_2_/O_2_ etching process (0–180 s), and the results are shown in Figure 7. Based on the test data, the elemental changes can be roughly divided into two distinct stages. In the first stage (the first 30 s of etching), there were significant changes in atomic concentrations. During this stage, the atomic percentage of oxygen in the Y_2_O_3_ coating increased sharply from 35% to 53%, and the atomic percentage of fluorine increased from 8% to 29%. For the YOF series coatings, the atomic percentage of fluorine also showed significant changes, with YOF 3%, YOF 6%, and YOF 9% coatings increasing from 18%, 10%, and 5% to 45%, 30%, and 19%, respectively. At the same time, the atomic percentage of oxygen in the YOF coatings decreased from 25%, 23%, and 39% to 19%, 21%, and 43%, respectively. These changes indicate that fluorine and oxygen underwent different dynamic changes during the etching process, with the fluorine content increasing significantly, while the oxygen content decreased. In the second stage (etching time from 30 to 180 s), the atomic concentration changes of all coatings slowed down. This indicates that, as etching time increased, the chemical reactions on the coating surface gradually stabilized. During this stage, the YOF 6% coating exhibited the least change, indicating that it was the most stable during the etching process. This may be due to the composition or structure of this coating having better etch resistance under the etching conditions. Furthermore, the study found that the experimental powder did not contain carbon, but during the coating preparation process, carbon was mainly infiltrated into the sample randomly from the atmosphere. During etching, most of the carbon underwent oxidation, forming gaseous CO and CO_2_, while a small portion of the carbon was deposited as polymeric residues on the coating surface. This phenomenon further suggests that the chemical changes on the coating surface during etching involved not only oxygen and fluorine but may also be influenced by carbon contamination.

**Figure 8:**
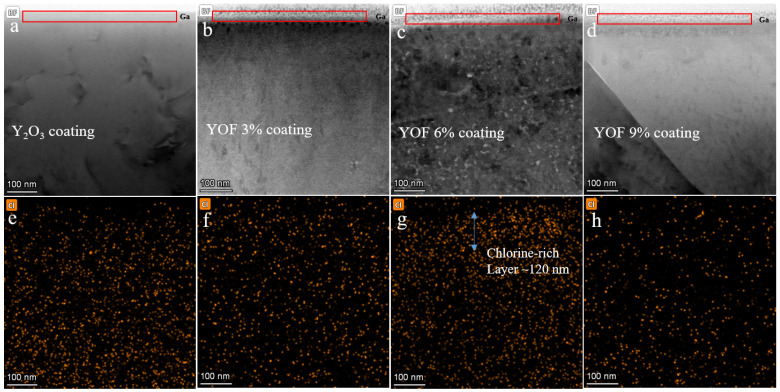
The TEM cross-sectional images of the (**a**) Y_2_O_3_ coating, (**b**) YOF 3% coating, (**c**) YOF 6% coating, and the (**d**) YOF 9% coating and the TEM-EDS mappings of chlorine in the (**e**) Y_2_O_3_ coating, (**f**) YOF 3% coating, (**g**) YOF 6% coating, and the (**h**) YOF 9% coating. The red box area in the figure is the Ga layer plated using the focused ion beam (FIB) method.

Figure 8a–d show cross-sectional TEM images of YOF and Y_2_O_3_ films after surface irradiation with Cl_2_ plasma, while Figure 8e–h present cross-sectional EDS mapping images of the same films after Cl_2_ plasma irradiation. The red box in the figures indicates the Ga layer plated using the focused ion beam (FIB) method. Analysis of these images reveals significant differences in the distribution of the chlorine element across the coatings. In particular, as shown in Figure 8g, the chlorine element is most densely distributed on the surface of the YOF 6% coating, with most of it being concentrated on the upper surface, forming a chlorine-rich layer approximately 120 nm thick. This suggests that the improved etching resistance of the YOF 6% coating may be closely related to the accumulation of chlorine ions on its surface. Chlorine ions can replace oxygen and fluorine ions in the coating, forming a protective layer that effectively prevents further chlorine ion erosion. In contrast, the chlorine element concentration on the surfaces of the Y_2_O_3_, YOF 3%, and YOF 9% coatings after etching is relatively low, and the distribution of chlorine is less uniform compared with the YOF 6% coating. This makes it more difficult for these coatings to form an effective protective layer against chlorine ions, resulting in poorer etching resistance in Cl_2_ plasma environments. Overall, the YOF 6% coating, due to its unique chlorine ion accumulation characteristic, exhibits the best etching resistance, while the other coatings, with uneven or low chlorine distribution, show relatively poor etching resistance.

## 4. Conclusions

YOF coatings with different oxygen contents were prepared on aluminum substrates using APS technology, and Y_2_O_3_ coatings were prepared for comparison. The etching resistance of all coatings in the Cl_2_/O_2_ plasma etching process was investigated. X-ray diffraction (XRD) results showed that after plasma etching, the diffraction peaks of YOF coatings shifted significantly to the left, indicating a change in lattice constants. This suggests that chlorine ions replaced fluorine and oxygen ions in the coating during the etching process, resulting in changes to the lattice structure. Scanning electron microscope (SEM) observations revealed that, compared with Y_2_O_3_ coatings, YOF series coatings exhibited denser microstructures and lower surface roughness after etching, demonstrating the superior etching resistance of YOF coatings. Etching depth measurements showed that the 6% YOF coating had the shallowest etching depth, only 6.342 µm, and the smallest mass loss per unit area, only 0.018 g/cm^2^, indicating the best etching resistance of this coating. Transmission electron microscope-energy dispersive spectroscopy (TEM-EDS) results showed that chloride ions accumulated on the surface of YOF series coatings, forming a chloride-rich layer. Among all the coatings, the chloride-rich layer of the 6% YOF coating was the thickest, reaching about 120 nm. This result suggests that the accumulation of chloride ions on the coating surface forms a protective chloride-rich layer, which is crucial for enhancing the etching resistance of the 6% YOF coating. In conclusion, the 6% YOF coating exhibited superior etching resistance compared with Y_2_O_3_, 3% YOF, and 9% YOF coatings in the Cl_2_/O_2_ plasma etching process, mainly due to the formation of its chloride-rich layer. This study indicates that YOF coatings, particularly the 6% YOF coating, have the potential to serve as a protective barrier against damage during the Cl_2_/O_2_ plasma etching process.

## Figures and Tables

**Table 1:** Parameters of Y_2_O_3_ and YOF series powders.

Parameter	Y_2_O_3_	YOF 3%	YOF 6%	YOF 9%
Median particle size (D50, µm)	34.0	27.1	26.6	27.7
Angle of repose (^∘^)	33.5	33.7	31.4	30.6

**Table 2:** The mass variation of Y_2_O_3_,YOF 3%,YOF 6%, and YOF 9% coatings before and after etching.

	Y_2_O_3_	YOF 3%	YOF 6%	YOF 9%
Pre-etching (g)	24.19	25.33	25.38	25.32
Post-etching (g)	23.74	24.54	25.11	24.59
Mass variation (g)	−0.45	−0.79	−0.27	−0.73
Decrement (g/cm^2^)	0.046	0.127	0.018	0.117

## Data Availability

The original contributions presented in this study are included in the article. Further inquiries can be directed to the corresponding author.
